# Congenital Hypertrophic Stenosis of the Pylorus

**Published:** 1903-11-14

**Authors:** 


### CONGENITAL HYPERTROPHIC STENOSIS OF THE PYLORUS.

It is becoming evident that this complaint is not so exceedingly rare as it was supposed to be a short while ago, and, since the condition is amenable to treatment, and the diagnosis is not always difficult, it is highly important for every medical man to be in a position to recognise cases when they occur. Mr. Moynihan[1] points out that the symptoms of the disease have been strikingly similar in the majority of the recorded cases. In a typical instance the child at birth is a well-nourished, healthy baby of normal weight. Soon after birth—it may be within the first few days or perhaps not until the baby is a few weeks old—it is noticed that the child begins to vomit after its food. At first it is only after the completion of a meal, or toward the end, that the food is returned. The quantity brought up is small at first but it increases day by day. It may then be observed that if only a little food be taken it is retained whereas a larger quantity is at once rejected. An alteration of the food from breast milk to peptonised milk or to diluted cows' milk is of little avail in checking the vomiting, or if there does appear to be any improvement it is only a temporary one. As time goes by the quantity of food that can be retained becomes less and less until at last nothing at all can be kept down. Bile is never present in the vomit. When the vomiting is thoroughly established its violence is remarkable. With a continuance of the symptoms the child rapidly wastes, the temperature is constantly subnormal, little or no motion is passed because the bowels are never filled. The mouth and tongue remain clean and moist as a rule. If the abdomen be examined after the symptoms have continued for some days, the outline of the dilated stomach may be clearly seen, and sometimes peristaltic waves are visible passing along it from left to right. A tumour may be palpable at the pylorus. The remainder of the abdomen is sunken and hollowed owing to the emptiness of the intestines, forming a marked contrast with the bulging out of the upper part by the dilated stomach.

Post-mortem specimens show that there is a great hypertrophy of the circular muscle fibres in the pyloric region, and in many cases the obstruction is so complete that water pumped into the stomach cannot be made to pass into the duodenum. No quite satisfactory explanation has been given of the etiology. By some it is thought that the muscular hyperplasia is primary, that is to say purely developmental in origin, while others hold that it is due to spasm leading to over-growth, and it has been argued that this spasm may commence in utero, being set up by the swallowing of amniotic fluid by the fœtus. With regard to treatment, Mr. Moynihan states that in the early stages of the disease before the vomiting has become incessant, much good may be done by careful washing out of the stomach, and feeding in small quantities through an indiarubber catheter. Undoubted instances have been recorded of recovery under purely medical treatment, but in the majority of cases where the symptoms have become well-established an operation will be required. The following methods have been carried out with varying success: dilatation of the pylorus, pyloroplasty and gastro-enterostomy. Each procedure has had its successes and its failures, and it remains for the future to decide which operation is the best.

[1] Medical News, Oct. 24.

